# Biweekly Versus Monthly Hyperimmune Globulin Therapy for Primary Cytomegalovirus Infection in Pregnancy

**DOI:** 10.3390/jcm12216776

**Published:** 2023-10-26

**Authors:** Nawa Schirwani-Hartl, Pilar Palmrich, Christina Haberl, Nicole Perkmann-Nagele, Herbert Kiss, Angelika Berger, Judith Rittenschober-Böhm, Gregor Kasprian, Patric Kienast, Asma Khalil, Julia Binder

**Affiliations:** 1 Department of Obstetrics and Gynecology, Division of Obstetrics and Feto-Maternal Medicine, Comprehensive Center for Pediatrics, Medical University of Vienna, 1090 Vienna, Austria; nawa.schirwani-hartl@meduniwien.ac.at (N.S.-H.); pilar.palmrich@meduniwien.ac.at (P.P.); christina.haberl@meduniwien.ac.at (C.H.); herbert.kiss@meduniwien.ac.at (H.K.); 2 Department of Laboratory Medicine, Medical University of Vienna, 1090 Vienna, Austria; 3 Department of Pediatrics and Adolescent Medicine, Division of Neonatology, Pediatric Intensive Care and Neuropediatrics, Comprehensive Center for Pediatrics, Medical University of Vienna, 1090 Vienna, Austria; angelika.berger@meduniwien.ac.at (A.B.); judith.rittenschober-boehm@meduniwien.ac.at (J.R.-B.); 4 Department of Radiology, Division of Neuroradiology and Musculoskeletal Radiology, Medical University of Vienna, 1090 Vienna, Austria; gregor.kasprian@meduniwien.ac.at (G.K.); patric.kienast@meduniwien.ac.at (P.K.); 5 Fetal Medicine Unit, St George’s University Hospitals NHS Foundation Trust, University of London, London WC1E 6BT, UK; asma.khalil@stgeorges.nhs.uk

**Keywords:** cytomegalovirus in pregnancy, hyperimmune globulin therapy, congenital infection, prenatal ultrasound, fetal magnetic resonance imaging, maternal–fetal transmission

## Abstract

Primary cytomegalovirus (CMV) infection during pregnancy is associated with an increased risk of congenital CMV (cCMV). Hyperimmune globulin (HIG) therapy has been proposed as a potential prophylaxis to reduce maternal–fetal transmission. Data on whether the administration of HIG every 2 weeks offers benefits over HIG administration every 4 weeks are lacking. This was a retrospective analysis including pregnant women with primary CMV infection diagnosed in the first or early second trimester between 2010 and 2022 treated with HIG every 4 weeks (300 IE HIG per kg) or every 2 weeks (200 IE HIG per kg), respectively. In total, 36 women (4 weeks: *n* = 26; 2 weeks: *n* = 10) and 39 newborns (4 weeks: *n* = 29; 2 weeks: *n* = 10) were included. The median gestational age at the first HIG administration was 13.1 weeks. There was no significant difference in the cCMV rates between the women who received HIG every 4 versus every 2 weeks (*n* = 8/24 [33.3%] vs. 3/10 [30.0%]; *p* = 0.850). An abnormal fetal ultrasound was present in three fetuses and fetal magnetic resonance imaging (MRI) anomalies in four fetuses were related to cCMV infection, with no significant difference in the frequency between the two groups. A larger study will be needed to determine whether HIG administration every 2 instead of every 4 weeks improves the maternal–fetal transmission rates.

## 1. Introduction

Cytomegalovirus (CMV) is the most common viral cause of intrauterine infections with a prevalence of 0.2–2.2% of all live births and a high number of unreported cases due to the lack of national screening programs [1,2,3]. The highest CMV disease burden is seen following congenital CMV (cCMV) infection with intrauterine transmission or transplacental transfer [4,5,6,7]. After a primary maternal CMV infection, the frequency of maternal–fetal transmission increases with the gestational age and ranges from 33% to up to 75%, as IgG transport increases with advancing gestational age [8]. However, previous studies suggest that the highest prevalence of symptomatic neonates at birth is seen with maternal seroconversion in the first trimester of pregnancy [9,10,11,12]. Severe complications such as sensorineural hearing loss, brain anomalies with neurodevelopmental delay, and seizures affect up to 10–15% of neonates [13]. Other symptoms of cCMV infection include microcephaly, hepatosplenomegaly, intrauterine growth restriction, as well as blindness [14,15,16,17,18]. Due to the increased risk of symptomatic cCMV infection, especially in cases of maternal–fetal transmission in the first trimester, management strategies to avoid transplacental CMV transmission including the administration of HIG have been introduced. Nigro et al. reported that HIG, which is manufactured from the plasma of elected high anti-CMV antibody avidity and titers, is a potential prenatal therapy for cCMV infection [19]. Several studies showed promising results for HIG as a therapeutic agent for preventing antenatal CMV transmission [19,20,21,22,23]. In contrast, two randomized controlled trials failed to show a significant effect of HIG therapy on transmission rates in pregnant women with a primary CMV infection [24,25]. A modified approach of administering HIG in biweekly intervals instead of every 4 weeks has been shown to reduce maternal–fetal transmission rates substantially [26].

The aim of this study was to evaluate the preventive effects against maternal–fetal transmission of CMV between HIG administration every 2 weeks and every 4 weeks following maternal primary CMV infection in the first or early second trimester of pregnancy.

## 2. Materials and Methods

This is a retrospective cohort study conducted at the Department of Obstetrics and feto-maternal Medicine at the Medical University of Vienna including all pregnant women treated with HIG following maternal primary CMV infection between January 2010 and September 2022. Women were referred to the Fetal Medicine Unit of the Medical University of Vienna for suspected primary CMV infection after being tested locally by their obstetrician or general health care practitioner when presenting with symptoms of CMV infection or due to being part of a high-risk group for CMV exposure. To date, no national screening program for CMV infection has been introduced in Austria.

From 2010 to 2018, women with a serologically confirmed primary CMV infection were offered to be treated with a protocol of 300 IE HIG per kg every 4 weeks (Cytotect, Frankfurt, Germany). In 2018, the local protocol was changed according to the recommendations by Kagan et al. [16] and pregnant women were treated with 200 IE HIG per kg maternal body weight biweekly (Cytotect, Frankfurt, Germany). Women included in this study were divided into two groups: biweekly versus every 4 weeks administrations. The primary outcome was maternal–fetal CMV transmission rate assessed by urine CMV PCR of the newborn compared between the two groups.

The inclusion criteria were as follows: confirmed primary CMV infection diagnosed in the first or early second trimester of pregnancy. Written and oral informed consent about the off-label use of HIG was obtained by each patient after detailed counseling about CMV infection in pregnancy by a fetal medicine consultant.

Clinical characteristics including maternal age, maternal height and weight, body mass index (BMI), mode of conception, parity, gestational age (GA) at the time of diagnosis, GA at the time of first HIG administration, GA at the time of birth, pregnancy outcome (e.g., mode of birth, live birth, intrauterine death) as well as the number of HIG administrations per patient were extracted from the obstetric electronic database (Viewpoint 5.6.8.428, Wessling, Germany). Moreover, newborn characteristics including sex, birth weight, admission to newborn intensive care unit (NICU), cord blood CMV PCR, postnatal antiviral therapy, urine CMV PCR, abnormal cerebral ultrasound, abnormal hearing assessment and abnormal eye examination were recorded.

Laboratory assessment was conducted at the Department of Laboratory Medicine at the Medical University of Vienna and maternal primary CMV infection was defined as follows: positive CMV immune globulin (Ig) M and positive CMV IgG with a low CMV IgG avidity or positive CMV desoxyribonucleic acid quantitative polymerase chain reaction (PCR) in maternal blood.

Fetal infection was diagnosed by amniotic fluid CMV PCR from amniocentesis at 20–21 weeks of gestation, which was recommended to all study participants and conducted after obtainment of patients’ oral and written informed consent. CCMV was confirmed by CMV PCR analysis of urine samples of the newborn after birth. Quantitative real time PCR from serum, EDTA-blood, amniotic fluid, and urine samples were assessed using NeuMoDx^TM^ CMV Quant Strip Test (NeuMoDx^TM^ Molecular, Qiagen, Germany), Roche LightCycler CMV Quant Kit on the LightCycler 480 platform (Roche Diagnostics, Rotkreuz, Switzerland) and Abbott RealTime CMV assay on the m2000 platform (Abbott Molecular, Des Plaines, IL, USA).

All patients underwent at least standardized detailed ultrasound assessments every 4 weeks by a fetal medicine specialist at the Fetal Medicine Unit of the Medical University of Vienna. Abnormal ultrasound findings were recorded. Fetal MRI was offered additionally to fetal ultrasound in the third trimester of pregnancy. Ingenia 1.5 Tesla (Philips Medical Systems, Best, The Netherlands) was used for fetal MRI.

Postnatal neonatal assessment included cerebral ultrasound, hearing screening (using brain evoked response auditory (BERA), eye examination and detailed neurological assessment. Additional cerebral MRI was performed if clinical abnormalities related to CMV infection were detected on cerebral ultrasound. Neonates with confirmed cCMV infection were evaluated six months, one year, and up to two years after birth.

This study was approved by the Ethics Committee of the Medical University of Vienna (2064/2020) and performed in accordance with the principles of Good Clinical Practice (GCP). Due to the retrospective design of the study, the Ethics Committee waived the need for written informed consent.

Continuous data were reported as median and interquartile range (IQR). Categorical variables were presented as number (*n*) and percentage (%) of patients with the characteristic of interest. As for comparisons of continuous variables between two groups, an unpaired *t*-test was applied. The Mann–Whitney U test was performed to compare continuous variables without normal distribution between the two groups. Group comparisons of categorical variables were performed using Pearson’s Chi-squared test. IBM SPSS 25.0 statistic software (IBM, Armonk, NY, USA) and GraphPad Prism 8 (Graphpad Software, La Jolla, CA, USA) were used for statistical analysis. A two-sided *p*-value of *p* < 0.050 was valued as statistically significant.

## 3. Results

### 3.1. Study Population

**Figure 1:**
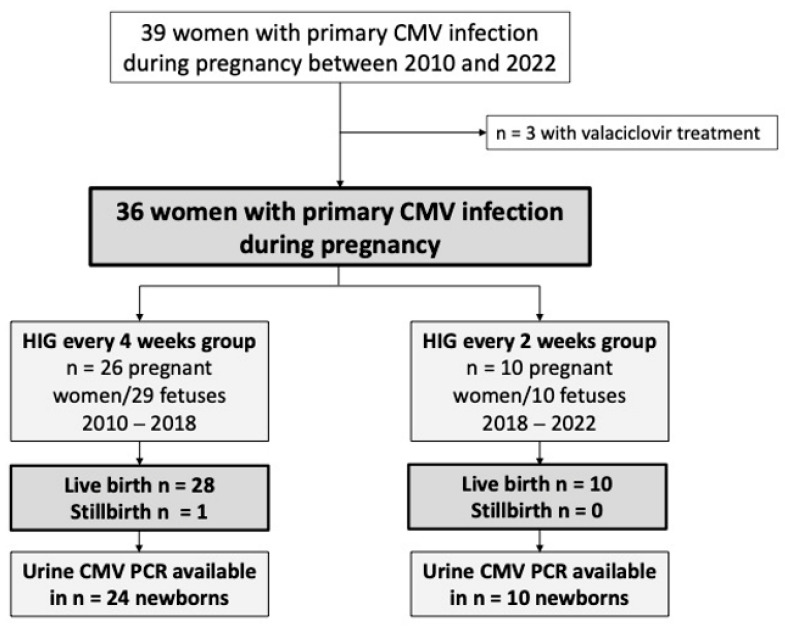
Flowchart of pregnant women with primary cytomegalovirus (CMV) infection and their fetuses/newborns included in this study.

The study population consisted of 39 pregnant women who presented with a primary CMV infection, of which three women received additional treatment with valaciclovir, and were therefore excluded from this analysis. A total of 36 pregnant women with a primary CMV infection (4 weeks: *n* = 26; 2 weeks: *n* = 10), who gave birth to 39 children (4 weeks: *n* = 29; 2 weeks: *n* = 10) fulfilled the inclusion criteria and were assessed in this study. Figure 1 depicts the cohort building process of the study population.

There were no significant differences between the two groups in terms of the maternal age (*p* = 0.710), BMI (*p* = 0.236), parity (*p* = 0.836), or mode of conception (*p* = 0.739), as presented in Table 1.

The median GA at the first presentation with a suspected primary CMV infection was 11.6 weeks; (IQR 8.6–14.0) and did not differ significantly between the groups (4 weeks: median 11.8 weeks [IQR 7.6–17.8] versus 2 weeks: median 11.1 weeks [IQR 8.6–12.7]; *p* = 0.471). The median GA at the first HIG administration was 13.5 weeks (IQR 12.4–19.8) in the HIG every 4 weeks group compared to 11.6 weeks (IQR 9.4–13.4) in the biweekly group (*p* = 0.063).

In total, 12 women opted for amniocentesis (4 weeks: *n* = 2 (16.7%) versus 2 weeks: *n* = 10 (100%), *p* < 0.001). The details concerning the amniocentesis cohort are given in Table 2.

### 3.2. HIG Administration

The median time from the diagnosis to first HIG administration did not differ between the two groups (4 weeks: 6.5 days [4.0–39.2] versus 2 weeks: 3.5 days [1.8–7.0]; *p* = 0.362).

### 3.3. Virological Data of Pregnant Women with Primary CMV Infection

In the entire cohort, the median maternal serum CMV viral load at the time of the first presentation at our center was 788.0 copies/mL [IQR 218.0–1700.0 copies/mL]. The women in the HIG every 4 weeks group had a significantly higher viral load in the maternal serum (4 weeks 1305.0 [IQR 424.5–3725.0 copies/mL] versus 174.0 [IQR 116.0–259.0 copies/mL] in the 2 weeks group (*p* = 0.006). Seven of the 36 (19.4%) patients tested negative for CMV DNA in serum/plasma (4 weeks: 19.2% compared to the 2 weeks: 20.0%). Twenty-nine (80.6%) women had detectable CMV DNA at the time of the first assessment. Table 3 shows the details of CMV-IgM, CMV IgG, CMV IgG avidity, and CMV PCR results of the women with a primary CMV infection.

### 3.4. Maternal–Fetal Transmission Rate

**Figure 2:**
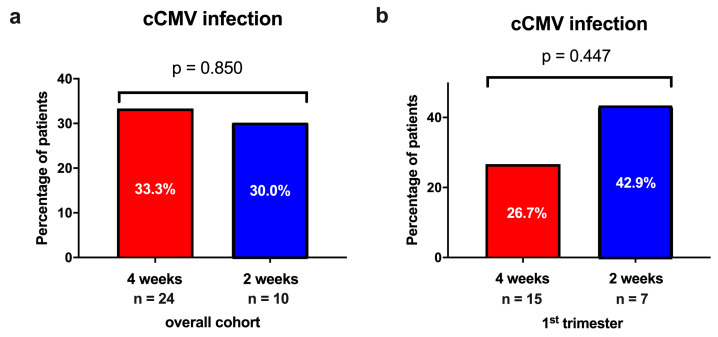
The incidence of congenital CMV (cCMV) infection stratified by the different hyperimmune globulin (HIG) treatment regimens (administration every 4 weeks vs. every 2 weeks) (**a**) in the overall cohort and (**b**) in newborns of women with primary CMV infection in the first trimester of pregnancy.

Overall, 11 (32.4%) neonates tested positive for cCMV infection confirmed by urine CMV PCR postnatally. There was no significant difference in the prevalence of cCMV infection between the newborns in the HIG every 4 weeks group versus the biweekly HIG group (4 weeks: *n* = 8/24 [33.3%] vs. 2 weeks: *n* = 3/10 [30.0%]; *p* = 0.850; Figure 2).

Four fetuses showed a positive CMV PCR in the amniotic fluid following an amniocentesis (4 weeks: *n* = 1 [50.0%] versus 2 weeks: *n* = 3 [30.0%]; *p* = 0.584) which was confirmed after birth. In the remaining eight cases with a PCR-negative amniotic fluid result, cCMV infection was again excluded at birth.

Similarly, when analyzing only the newborns of mothers with a primary CMV infection during the first trimester of pregnancy, the prevalence of cCMV infection in the neonates was not significantly different between the two groups (4 weeks: *n* = 4/15 [26.7%] vs. 2 weeks: *n* = 3/7 [42.9%]; *p* = 0.448).

### 3.5. Imaging Outcomes

All women regularly underwent a detailed fetal assessment by ultrasound and three fetuses (3/36; 8.3%) showed abnormalities (4 weeks: *n* = 2 [66.7%] versus 2 weeks: *n* = 1 [33.3%]; *p* = 0.751). One fetus in the HIG every 4 weeks group presented with fetal growth restriction and one with oligohydramnios due to a preterm premature rupture of membranes (PPROM). One fetus in the two weekly HIG group showed intraventricular adhesions on fetal ultrasound.

A total of 25 pregnant women underwent fetal MRI; four (4/25; 16.0%) presented with abnormalities (4 weeks: *n* = 4/20 [20.0%] vs. 2 weeks: *n* = 0/5 [0.0%]; *p* = 0.275). Table 4 shows details regarding the cases with fetal imaging abnormalities and clinical findings at birth.

In the group receiving HIG every 4 weeks, intracerebral abnormalities, placental edema, as well as splenomegaly were detected on fetal MRI. The fetal MRI abnormalities found in the biweekly HIG group were not specific for cCMV infection and no intracerebral lesions could be detected.

### 3.6. Neonatal Characteristics and Outcomes

The median birthweight, GA at delivery, mode of delivery, and NICU admission were not statistically different between the groups, as shown in Table 5.

**Figure 3:**
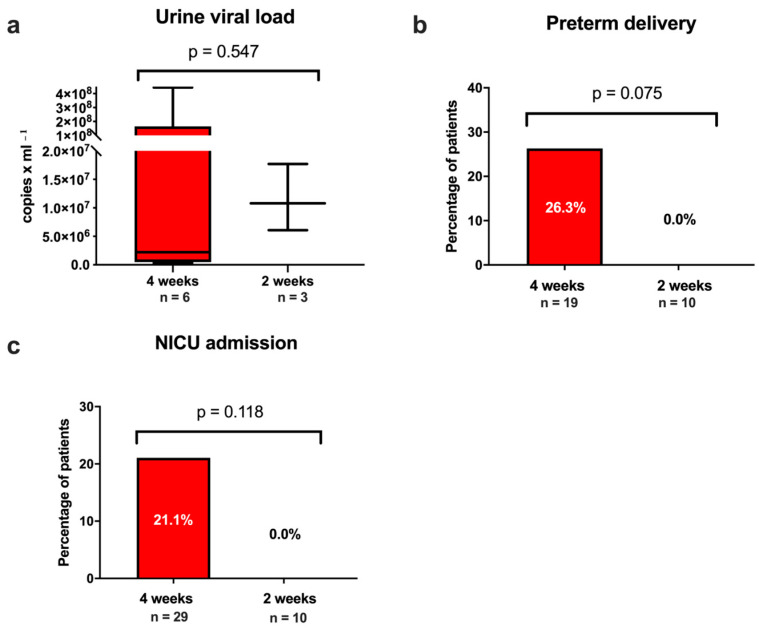
(**a**) Cytomegalovirus (CMV) viral load among newborns with mothers with primary CMV infection during pregnancy stratified for hyperimmune globulin treatment regimen (every 4 weeks, every 2 weeks). Prevalence of (**b**) preterm birth and (**c**) neonatal intensive care unit (NICU) admission. (**a**) Data presented as boxplots, the whiskers represent the minimum to maximum. Data provided as copies/mL.

As depicted in Figure 3, the comparison of the rates of preterm births between the HIG every 4 weeks and the two-weekly HIG group showed higher incidences in the every 4 weeks group, but did not reach statistical significance (4 weeks: *n* = 5/19 [26.3%] vs. 2 weeks: *n* = 0/10 [0.0%]; *p* = 0.075).

All neonates were liveborn except for one intrauterine fetal death (IUD) in the HIG every 4 week group (*n* = 1/29 [3.4%]), which was due to fetal growth restriction (<3 percentile), fetal inflammatory response syndrome, profound intracerebral lesions confirmed by MRI, and histologically confirmed large infarcts in the placenta.

Three neonates with confirmed cCMV infection showed abnormal clinical findings at birth (4 weeks: *n* = 3/24, [12.5%] versus 2 weeks: *n* = 0/10 [0.0%]; *p* = 0.242) related to cCMV infection such as intracranial cystic lesions. A proportion of 2/13 newborns (15.4%) receiving postnatal cerebral ultrasound had imaging abnormalities (4 weeks: *n* = 2/8 [25.0%] versus 2 weeks *n* = 0/5 [0.0%]; *p* = 0.224), such as periventricular hyperechogenicity.

The median urine viral load among the neonates with cCMV infection was 6.1 × 10^6^ copies/mL (IQR: 9.5 × 10^5^–4.4 × 10^7^ copies/mL) with no significant difference between the two groups (4 weeks: 2.2 × 10^6^ [IQR 4.9 × 10^5^–1.6 × 10^8^ copies/mL] versus 2 weeks: 1.1 × 10^7^ [IQR 6.1 × 10^6^–1.7 × 10^7^ co/mL]; *p* = 0.547).

## 4. Discussion

This study compared the maternal–fetal CMV transmission rates in 36 pregnant women with a primary CMV infection in the first and early second trimester of pregnancy receiving HIG therapy either every 4 weeks or biweekly. In both cohorts, the rate of cCMV infection was high (33.3% in the every 4 weeks group vs. 30.0% in the biweekly group). There was no significant difference in the maternal–fetal transmission rates between the two groups.

Previous studies have shown CMV transmission rates of 30–50% following maternal primary CMV infection in the first trimester [27,28], potentially leading to long-term complications and life-threatening conditions in the offspring [27]. HIG has been suggested as a therapeutic option for women with primary CMV infection in the first trimester of pregnancy. A prospective observational study using a historical control group found that biweekly HIG administration in early pregnancy was more effective in preventing maternal–fetal CMV transmission at up to 20 weeks of gestation (HIG: 7.5% vs. control: 35.2%, *p* < 0.001) [26]. Other studies indicated that HIG therapy may decrease the rate of maternal–fetal CMV transmission or the severity of symptoms caused by cCMV infection [19,21,22,23]. However, two randomized controlled trials failed to show an effect of HIG administration every 4 weeks on the transmission rates in pregnant women with a primary CMV infection [24,25]. As a result, HIG therapy is still not recommended as a standard therapy for primary CMV infection in pregnancy and can only be offered as an off-label use therapy or within clinical trials [29].

Our study was the first to compare HIG administration every 4 weeks with every 2 weeks in women with a primary CMV infection during the first and early second trimester of pregnancy. Assessing urine samples for CMV PCR postnatally, we found high rates of cCMV infection ranging from 30–33.3% in both treatment groups, despite HIG administration. This is consistent with the current literature describing cCMV rates of 30% to 66% in neonates of pregnant women treated with HIG [23,24]. Importantly, no difference in the incidence of cCMV infection was observed between the groups treated with HIG biweekly and every four weeks, indicating that HIG administration in shorter time intervals is not associated with higher rates of prevention of maternal–fetal CMV transmission. Even when analyzing women with cCMV infection in the first trimester separately, biweekly HIG administration was not superior to HIG administration every four weeks. The transmission rates observed in both groups are similar to the above-mentioned vertical transmission rates of CMV without therapy [27,28], suggesting that HIG might not be effective in reducing vertical CMV transmission. However, none of the newborns included in this study exhibited severe sequelae of cCMV compared to 10–15% described in the literature [13], suggesting a potential effect on the disease severity of HIG administration.

In contrast to our findings, Kagan et al. observed an overall transmission rate of cCMV of 7.5% after following a biweekly HIG regimen in women with a confirmed cCMV infection in the first trimester [26]. However, the management protocol only allowed a very narrow window of opportunity to treat women for cCMV infection as well as required the extensive and repeated testing of CMV avidity and reactivity. Both requirements question the practicability and cost effectiveness in a routine clinical setting without a national screening program in place.

However, our data indicated the potential benefits of biweekly HIG administration over administration every 4 weeks concerning the neonatal outcome. The neonates of women treated with biweekly HIG were less frequently born preterm and admitted to NICU. Moreover, neonates in the HIG every 4 weeks group more frequently presented with severe findings on fetal imaging and at assessment at birth such as cerebral abnormalities, fetal inflammatory response syndrome and stillbirth, although the numbers did not reach statistical significance. This might support the findings of previous studies suggesting the advantageous effects of HIG administration [21,23,25,26], and indicate that shorter HIG administration intervals might be more efficient due to the half-life of HIG of around 11 days.

This is the first study comparing biweekly to HIG administration every 4 weeks in pregnant women with cCMV infection in a routine clinical setting over a 12-year (HIG every 4 week group) or 4-year period (HIG every 2 week group), respectively. Detailed assessment of the virological, clinical and imaging data of the study participants as well as a long-term follow up of their infants of up to two years was conducted and is described in this study. However, our study has some limitations. Firstly, we acknowledge the small sample size within the treatment groups, which might have limited the ability to detect small effects of HIG in our cohort and is due to the retrospective study design. The limited power represents the most important limitation of this study. Secondly, protocols for HIG administration were changed within the study period and dosages were adapted from 300 IE every four weeks to 200 IE every two weeks. Additionally, the time from diagnosis of primary CMV infection to initiation of HIG treatment was longer—albeit non-significantly—in the HIG every 4 weeks group. Interestingly, even with a numerically longer time period between the first diagnosis and HIG administration in the HIG every 4 weeks group as well as a longer time period between HIG administration every 4 instead of every 2 weeks, there was no difference in the transmission rates between the groups. Finally, further limitations of this study include the lack of a non-treated control group and the smaller sample size of the HIG every 2 weeks group, which is attributable to the retrospective nature of this study.

HIG administration is considered to be relatively safe in pregnancy. However, a significant reduction in the maternal–fetal CMV transmission rate could not be observed in a routine clinical setting. Our data suggest a beneficial effect of biweekly HIG administration over HIG administration every 4 weeks on the long-term morbidity in infants with cCMV. The study cohort, however, was too small to generate results of statistical significance. Randomized controlled trials would be necessary to address this research question.

## 5. Conclusions

Our study showed no difference in the maternal–fetal CMV transmission rates between pregnant women with a primary CMV infection receiving HIG every 4 weeks versus every 2 weeks. The incidence of cCMV infections was high in both groups, suggesting that HIG treatment is not effective in preventing maternal–fetal transmission. However, more frequent HIG administration might provide beneficial effects on the neonatal outcome. Randomized controlled trials would be needed to further explore this.

## Figures and Tables

**Table 1:** Patient characteristics of pregnant women with primary cytomegalovirus infection included in this study (*n* = 36) and comparison between women treated with hyperimmune globulin (HIG) every 4 weeks (4 weeks) and every 2 weeks (2 weeks). Data presented as median and interquartile range (IQR) or number (%).

Parameters	All Patients (*n* = 36)	HIG Every 4 Weeks (*n* = 26)	HIG Every 2 Weeks (*n* = 10)	*p*-Value
Maternal age, years	30.5 (27.4–34.8)	31.9 (26.9–35.6)	31.5 (28.3–34.3)	0.710
Maternal body mass index, kg/m^2^	21.9 (20.2–26.3)	22.5 (21.3–27.6)	21.1 (19.0–22.2)	0.236
Nullipara, *n* (%)	19 (52.8%)	14 (53.8%)	5 (50.0%)	0.836
Mode of conception, *n* (%)				0.739
Spontaneous	33 (91.6%)	23 (88.6%)	10 (100.0%)	
In vitro fertilization	1 (2.8%)	1 (3.8%)	0 (0.0%)	
Intracytoplasmic sperm injection	1 (2.8%)	1 (3.8%)	0 (0.0%)	
Stimulation	1 (2.8%)	1 (3.8%)	0 (0.0%)	
Median gestational age at first diagnosis, days (IQR)	11.6 (8.6–14.0)	11.8 (7.6–17.8)	11.1 (8.6–12.7)	0.471
Median gestational age at first HIG administration, days (IQR)	13.1 (11.9–17.0)	13.5 (12.4–19.8)	11.6 (9.4–13.4)	0.063
Median time between first diagnosis and administration, days	5.0 (3.0–13.0)	6.5 (4.0–39.2)	3.5 (1.8–7.0)	0.362

**Table 2:** Characteristics of women undergoing amniocentesis (*n* = 12). Data presented as median and IQR or as number (%).

	All Patients *n* = 12	HIG Every 4 Weeks *n* = 2	HIG Every 2 Weeks *n* = 10	*p*-Value
Gestational age at amniocentesis, weeks (IQR)	21.4 (20.6–21.8)	23.9 (n.a.)	21.4 (20.7–21.5)	0.203
Time interval between first presentation and amniocentesis, weeks (IQR)	11.0 (8.0–12.0)	11.5 (n.a.)	9.0 (7.5–12.0)	0.999
Materno-fetal transmission rate at time of amniocentesis, *n* (%)	4 (33.3%)	1 (50.0%)	3 (30.0%)	0.584

n.a. = not applicable.

**Table 3:** Virology data of pregnant women with primary cytomegalovirus (CMV) infection obtained from maternal blood. Data presented as median and interquartile range (IQR) or as number (%).

Parameters	All Patients (*n* = 36)	HIG Every 4 Weeks (*n* = 26)	HIG Every 2 Weeks (*n* = 10)	*p*-Value
CMV DNA qPCR				
Not detectable at first presentation, *n* (%)	7 (19.4%)	5 (19.2%)	2 (20.0%)	0.958
Detectable at first presentation, *n* (%)	29 (80.6%)	21 (80.8%)	8 (80.0%)	
CMV viral load in cases with detectable CMV DNA qPCR, co/mL	788.0 (218.0–1700.0)	1305.0 (424.5–3725.0)	174.0 (116.0–259.0)	0.006
CMV -IgM at first presentation, *n* (%)				
CMV IgM negative	1 (2.8%)	0 (0.0%)	1 (10.0%)	0.222
CMV IgM positive	34 (94.4%)	25 (96.2%)	9 (90.0%)	
CMV IgM borderline	1 (2.8%)	1 (3.8%)	0 (0.0%)	
CMV IgG at first presentation, *n* (%)				
CMV IgG negative	0 (0%)	0 (0%)	0 (0%)	0.491
CMV IgG positive	36 (100%)	26 (100%)	10 (100%)	
CMV IgG avidity at first presentation, *n* (%)				
Low avidity	30 (83.3%)	22 (84.6%)	8 (80.0%)	0.679
Borderline avidity	5 (13.9%)	3 (11.6%)	2 (20.0%)	
Seroconversion	1 (2.8%)	1 (3.8%)	0 (0.0%)	

**Table 4:** Overview of clinical cases of newborns with cCMV infection. This table lists all newborns with cCMV infection. The cases of imaging abnormalities via ultrasound or magnetic resonance imaging (MRI) detected abnormalities as well as the clinical findings at birth of newborns with cCMV.

Case	GA ^1^ at Diagnosis in Weeks	Fetal Ultrasound Abnormalities	Fetal MRI ^2^ Abnormalities	HIG Interval	Findings at Birth	Hearing Test, Eye Examination, Neurological Outcome Two Years after Birth
1	17.1	-	Small temporal cysts	Every 4 weeks	Intracerebral cystic lesions at neonatal ultrasound	No abnormalities
2	12.9	Oligohydramnios	-	Every 4 weeks	pPROM ^3^ at 34 + 2 weeks, preterm delivery in 34 + 4 weeks	No abnormalities
3	11.0	Fetal growth retardation (<3. percentile)	Splenomegaly, subependymal cysts, leucencephalopathy, placental edema	Every 4 weeks	Stillbirth at 40.0 weeks, MRI ^2^: profound intracerebral lesions Histology: large infarcts in the placenta	-
4	11.6	Intraventricular adhesion	-	Every 2 weeks	Asymptomatic, normal BERA ^4^, eye exam, neurological exam, and neurological development	No abnormalities
5	11.6	-	Placental anomalies in correlation with fetal inflammatory response syndrome	Every 4 weeks	Asymptomatic, normal BERA ^4^, eye exam, neurological exam, and neurological development	No abnormalities
6	22.7	-	Second MRI†: Bilateral small cysts in the medial temporal lobe	Every 4 weeks	Asymptomatic, normal BERA ^4^, eye exam, neurological exam, and neurological development	No abnormalities
7	7.3	-		Every 4 weeks	Asymptomatic, normal BERA ^4^, eye exam, neurological exam, and neurological development	No abnormalities
8	13	-		Every 4 weeks	Asymptomatic, normal BERA ^4^, eye exam, neurological exam, and neurological development	No abnormalities
9	8.6	-		Every 2 weeks	Asymptomatic, normal BERA ^4^, eye exam, neurological exam, and neurological development	No abnormalities
10	9.9	-		Every 2 weeks	Asymptomatic, normal BERA ^4^, eye exam, neurological exam, and neurological development	No abnormalities
11	23.6	-		Every 4 weeks	Asymptomatic, normal BERA ^4^, eye exam, neurological exam, and neurological development	No abnormalities

^1^ Gestational age. ^2^ Magnetic resonance imaging. ^3^ Premature rupture of membranes. ^4^ Brain evoked response auditory.

**Table 5:** Pregnancy and neonatal outcomes. Data presented as median and interquartile range (IQR) or as number (%).

Parameters	All Patients (*n* = 39)	HIG Every 4 Weeks (*n* = 29)	HIG Every 2 Weeks (*n* = 10)	*p*-Value
Sex, *n* (%)				0.313
Male	17 (34.6%)	11 (37.9%)	6 (60.0%)	
Female	18 (46.1%)	14 (48.3%)	4 (40.0%)	
Unknown	4 (10.3%)	4 (13.8%)	0 (0.0%)	
Singleton pregnancy, *n* (%)	33 (84.6%)	23 (79.3%)	10 (100.0%)	0.118
Preterm birth < 37 weeks, *n* (%)	5/29 (17.2%)	5/19 (26.3%)	0/10 (0.0%)	0.075
Gestational age at delivery, weeks	38.9 (36.6–40.0)	38.6 (36.3–40.0)	39.4 (38.2–40.3)	0.734
Birthweight, grams	3440.0 (2955.0–3655.0)	3460.0 (2710.0–3760.0)	3365.0 (3067.5–3507.5)	0.191
Mode of birth, *n* (%)				0.470
Vaginal delivery	16/30 (53.3%)	11/20 (55.0%)	5/10 (50.0%)	
ventouse	1/30 (3.3%)	0/20 (0.0%)	1/10 (10.0%)	
Elective cesarean section	12/30 (40.0%)	8/20 (40.0%)	4/10 (40.0%)	
Emergency cesarean section	1/30 (3.3%)	0/20 (0.0%)	1/10 (10.0%)	
Neonatal unit admission, *n* (%)	4/29 (13.8%)	4/19 (21.1%)	0/10 (0.0%)	0.118
Stillbirth, *n* (%)	1/39 (2.6%)	1/29 (3.4%)	0/10 (0.0%)	0.552
Congenital CMV infection, *n* (%)	11/34 (32.4%)	8/24 (33.3%)	3/10 (30.0%)	0.850
Urine CMV viral load in cases with detectable CMVDNA qPCR	6.1 × 10^6^ (9.5 × 10^5^–4.4 × 10^7^)	2.2 × 10^6^ (4.9 × 10^5^–1.6 × 10^8^)	1.1 × 10^7^ (6.1 × 10^6^–1.7 × 10^7^)	0.547
Antibodies in cord blood, *n* (%)	8/14 (57.1%)	6/11 (54.5%)	2/3 (66.7%)	0.707

## Data Availability

The data are available upon reasonable request to the corresponding author.
